# Association between Atmospheric Fine Particulate Matter and Hospital Admissions for Chronic Obstructive Pulmonary Disease in Southwestern Taiwan: A Population-Based Study

**DOI:** 10.3390/ijerph13040366

**Published:** 2016-03-25

**Authors:** Su-Lun Hwang, Su-Er Guo, Miao-Ching Chi, Chiang-Ting Chou, Yu-Ching Lin, Chieh-Mo Lin, Yen-Li Chou

**Affiliations:** 1 Department of Nursing, Chang Gung University of Science and Technology, Chiayi Campus, Chiayi County 613, Taiwan; seguo@mail.cgust.edu.tw (S.-E.G.); ctchou@mail.cgust.edu.tw (C.-T.C.); f124510714@cgmh.org.tw (C.-M.L.); 2 Chronic Diseases and Health Promotion Research Center, Chang Gung University of Science and Technology, Chiayi County 613, Taiwan; mcchi@mail.cgust.edu.tw; 3 Department of Respiratory Care, Chang Gung University of Science and Technology, Chiayi County 613, Taiwan; lin0927@cgmh.org.tw; 4 Division of Pulmonary and Critical Care Medicine, Chang Gung Memorial Hospital, Chiayi County 613, Taiwan; mr2554@cgmh.org.tw; 5 Department of Respiratory Care, Chang Gung University, Taoyuan 333, Taiwan; 6 Graduate Institute of Clinical Medical Sciences, College of Medicine, Chang Gung University, Taoyuan 333, Taiwan

**Keywords:** fine particulate matter, chronic obstructive pulmonary disease, hospital admissions

## Abstract

*Objectives*: This paper reports on the findings of a population-based study to evaluate the relationship between atmospheric fine particulate matter (PM_2.5_) levels and hospital admissions for chronic obstructive pulmonary disease (COPD) in southwestern Taiwan over a three-year period, 2008–2010. *Methods*: Data on hospital admissions for COPD and PM_2.5_ levels were obtained from the National Health Insurance Research database (NHIRD) and the Environmental Protection Administration from 2008 to 2010, respectively. The lag structure of relative risks (RRs) of hospital admissions for COPD was estimated using a Poisson regression model. *Results*: During the study period, the overall average hospitalization rate of COPD and mean 24-h average level of PM_2.5_ was 0.18% and 39.37 μg/m^3^, respectively. There were seasonal variations in PM_2.5_ concentrations in southwestern Taiwan, with higher PM_2.5_ concentrations in both spring (average: 48.54 μg/m^3^) and winter (49.96 μg/m^3^) than in summer (25.89 μg/m^3^) and autumn (33.37 μg/m^3^). Increased COPD admissions were significantly associated with PM_2.5_ in both spring (February–April) and winter (October–January), with the relative risks (RRs) for every 10 μg/m^3^ increase in PM_2.5_ being 1.25 (95% CI = 1.22–1.27) and 1.24 (95% CI = 1.23–1.26), respectively, at a lag zero days (*i.e.*, no lag days). Lag effects on COPD admissions were observed for PM_2.5_, with the elevated RRs beginning at lag zero days and larger RRs estimates tending to occur at longer lags (up to six days, *i.e.*, lag 0–5 days). *Conclusions*: In general, findings reveal an association between atmospheric fine particulate matter (PM_2.5_) and hospital admissions for COPD in southwestern Taiwan, especially during both spring and winter seasons.

## 1. Introduction

Chronic obstructive pulmonary disease (COPD) has been reported to be a leading cause of morbidity and mortality and will become the third leading cause of death by 2030 worldwide [1]. COPD results in a substantial economic burden on healthcare services [2,3]. The National Heart Lung Blood Institute (NHLBI) estimated that the direct cost of COPD was $29.5 billion in the U.S. in 2010 [2]. In Singapore, the average direct cost of COPD was $9.9 million per year from 2005 to 2009 [3]. Several studies have reported that the morbidity and mortality rate of COPD is increasing in the Asia-Pacific regions [3,4,5]. In the Asia-Pacific, overall mortality and morbidity rates ranged 3.7–5.3 and 28.1–207.3 per 10,000 in 2003, respectively [5]. Furthermore, the overall prevalence rate of COPD in 12 Asia-Pacific countries was estimated by model with an overall prevalence rate of 6.3% (range 3.5%–6.7%) [6]. In Taiwan, chronic lower respiratory diseases ranked as the sixth leading cause of death in men (40.2 per 100,000) and the ninth in women (14.1 per 100,000) in 2012 [7], and the mortality of COPD was 17.88% per 100,000 in 2002 [8]. Moreover, the estimated average annual prevalence and incidence rates of chronic airway obstruction (CAO) were 2.48% and 0.66% from 1996 to 2002, respectively [9].

Epidemiological studies have reported that air pollution has been associated with excessive respiratory [10,11] and cardiovascular morbidity [11,12], as well as mortality [12]. Fine particulate matter (PM_2.5_, defined as particulate matter with an aerodynamic diameter less than 2.5 μm) present more potential threats to human health, because they can easily penetrate into the small airways and the alveoli of the lung [13]. Recently, exposure to PM_2.5_ has been associated with increased hospital admissions for multiple causes, including for all respiratory causes [12,13,14,15], COPD [10,12,16,17], cardiovascular disease [13,15] and stroke and diabetes [15,18] in North America, Europe and Hong Kong. Several meta-analysis studies have indicated that short-term exposure to ambient PM_2.5_ is associated with increased respiratory disease hospitalizations and mortality [19,20]. PM concentrations in Taiwan often exceed the recommended World Health Organization’s Air Quality guidelines [21]. However, epidemiological studies focused on the effects of ambient PM_2.5_ on COPD alone in Asia are limited. On the other hand, so far, studies on the lag effects and seasonal variability of PM_2.5_ on COPD are rare, and conflicting results have been reported in different countries and regions. Ko *et al.* [10] pointed out that ambient concentrations of air pollutants have an adverse effect on hospital admissions for COPD in Hong Kong, especially during the winter season. A study conducted in northern Taiwan reported that higher levels of PM_2.5_ may increase the risk of inpatient admissions for COPD, and the relationship between particulate matter (PM) and respiratory disease-related hospital admissions differed by season [22]. Chen *et al.* [23] found that higher levels of PM enhance the risk of hospital admissions for COPD only on cool days in Kaohsiung, in southern Taiwan. Moreover, the differential distributed lag patterns of PM on COPD hospitalizations had not been investigated in previous studies in Taiwan [22,23]. The seasonal variation in lag effects and effect estimates of PM_2.5_ on COPD could be explained by variation in exposure patterns and seasonal differences in the air pollution mixture [10,22,23].

Taiwan is located under the lee side of the East-Asian winter and summer monsoons, sea-land breezes (SLBs) and the Northeastern Monsoon (NEW), which play important roles in the recirculation and accumulation fine particulate matter over southern Taiwan [24,25]. In spring, the NEW often drives dust storms originating in the deserts of Mongolia and China (such as the Asian Dust Storm (ADS)) to Taiwan [26]. The ADS could lead to enhanced PM_2.5_ levels over the southwestern Taiwan. On the other hand, the Central Mountain Range is aligned north-south in Taiwan, and this can influence the geographical and meteorological conditionals in many locations throughout Taiwan, and might further affect the differences of air pollution in the recirculation and accumulation of air pollution over Taiwan. Yang and Kao [27] indicated that the PM_2.5_ annual mean concentrations ranged between 15.1 μg/m^3^ and 46.0 μg/m^3^ in Taiwan, and there are higher PM_2.5_ concentrations in southern Taiwan (annual mean concentrations increased above 40 μg/m^3^). The concentrations of PM_2.5_ in southwestern Taiwan were high in 2008–2010 compared to other administrative districts in Taiwan [22]. According to the Air Quality Annual Report of R.O.C. (Taiwan), it was indicated that the annual mean concentrations of PM_2.5_ in southwestern Taiwan in 2014 (ranging between 29.6 and 34.7 μg/m^3^) are higher than those in other areas in Taiwan (annual mean concentrations in northern, central and eastern Taiwan ranged 18.1–22.4, 23.8–29.8 and 11.3–13.7 μg/m^3^, respectively) [21]. However, the effects of PM_2.5_ on COPD have rarely been investigated in southwestern Taiwan. The objective of this study was to assess the impact of atmospheric PM_2.5_ levels on hospital admissions for COPD among residents in southwestern Taiwan, over a three-year period, 2008–2010, based on a population-based study.

## 2. Materials

### 2.1. Database

This study has been approved by the Institutional Review Board (IRB) of Chang Gung Medical Foundation (IRB No. 101-3077C). A population-based study was conducted using a subset of medical claims data from the National Health Insurance Research Database (NHIRD) obtained from the National Health Insurance (NHI) program in Taiwan (Registered Number: NHIR-NHIRD 101-533). The NHI program, which provides compulsory universal health insurance in Taiwan, has operated since 1995 and had an initial coverage rate of 90% of the Taiwanese population. At the end of 2008, over 99% of the Taiwanese population were enrolled in the NHI program [28]. The National Health Research Institutes in Taiwan has been responsible for managing the NHIRD. The NHIRD appears to be a valid resource for epidemiological research, with the information on prescription use, diagnosis and hospitalizations having been shown to be of high quality [29,30].

### 2.2. Study Population

Cases of COPD were ascertained by the service claim for patients who had at least one inpatient visit with a primary diagnosis of COPD (International Classification of Diseases, 9th revision, Clinical Modification (ICD-9-CM) Code 490, 491, 492 and 496). Hospitalization rates for men and women (and overall) population aged ≥40 years were estimated and adjusted by annual age-specific population. Daily counts of hospital admissions for COPD were extracted from the medical insurance file (NHIRD) for the period of 2008–2010. The analyses covered 4 municipalities (Chiayi City, Chiayi County, Tainan City and Tainan County), which are located in southwestern Taiwan. The inpatient visits were further analyzed by four seasons (spring as February, March and April; summer as May, June and July; autumn as August, September and October; winter as November, December and January) [11].

### 2.3. Outdoor PM_2.5_ Data

For each day, hourly PM_2.5_ concentrations data during 2008–2010 were obtained from the Environmental Protection Administration’s (EPA) Taiwan Air Quality Monitoring Network (TAQMN) [31]. According to geographical characteristics and air quality conditions, the TAQMN divides Taiwan into seven air quality regions, including Northern, Chu-Miao, Central, Yun-Chia-Nan, Kao-Ping, Yilan and Hua-Tung. The region of this study was located in the Yun-Chia-Nan air quality region, and eight ambient area air quality-monitoring stations (downwind or upwind of a high density of population) were located in four districts (Chiayi City, Chiayi County, Tainan City and Tainan County) distributed throughout southwestern Taiwan. However, PM_2.5_ was measured by seven stations only. The Chiayi station (23.27° N, 120.26° E), located in Chiayi City, is an urban site. The Xingan (23.33° N, 120.20° E) and Puzi stations (23.27° N, 120.14° E), located in Chiayi County, are rural sites. The Annan (23.02° N, 120.13° E) and Tainan stations (22.59° N, 120.12° E), located in Tainan City, are urban sites. The Xinying (23.18° N, 120.19° E) and Shanhua stations (23.06° N, 120.17° E), located in Tainan County, are rural sites. The 24-h average levels of PM_2.5_ were calculated from hourly PM_2.5_ concentrations and for further analysis.

### 2.4. Statistics

Hospitalization rates from 2008 to 2010 for the population were estimated and adjusted by annual age-specific population. Additionally, Poisson regression was performed to determine the association between COPD inpatient visits and the levels of PM_2.5_ using the relative risk (RR) and their 95% confidence intervals (95% CI). We examined lag effects of different days, including same-day lag (Lag 0); and cumulative lags by 2 (average of Lags 0 and 1), 3 (average of Lags 0, 1 and 2) to 6 days (average of Lags 0–5) [10,32]. The criterion for statistical significance was *p* < 0.05. Statistical analysis was performed using statistical software (SAS Version 8.2; SAS Institute; Cary, NC, USA).

## 3. Results

There were 9568 admissions for chronic obstructive pulmonary disease from January 2008 to December 2010 with an average annual hospitalization rate of 0.18% for the population aged ≥40 years in southwestern Taiwan, 0.26% for male and 0.10% for female. There were more male than female hospitalizations, with male-to-female ratios ranging from 2.00 to 3.50 by age (Table 1). Table 2 summarizes the means, standard deviations and percentiles of the daily counts of hospital admissions and the daily levels of PM_2.5_. During the study period, the daily average concentration for PM_2.5_ was 39.37 μg/m^3^. There was a mean of 8.73 daily hospital admissions for COPD in southwestern Taiwan over the study period.

Table 3 shows the seasonal concentrations of PM_2.5_ and the relative risks (RRs) estimates of PM_2.5_ on hospital admissions for COPD. There were obvious seasonal variations in PM_2.5_ in southwestern Taiwan, with higher PM_2.5_ concentrations in both spring (average daily mean concentrations: 48.54 μg/m^3^) and winter (49.96 μg/m^3^) than in summer (25.89 μg/m^3^) and autumn (33.37 μg/m^3^). In addition, COPD hospital admissions showed elevated relative risks (RRs) beginning at Lag 0 (*i.e.*, no lag days) and the highest RRs at Lags 0–5 (average of Lags 0–5, *i.e.*, cumulative lags by 6 days) in both spring and winter, with the RRs for every 10 μg/m^3^ increase in PM_2.5_ being 1.51 (95% CI = 1.46–1.55) and 1.54 (95% CI = 1.50–1.58), respectively. It seems, then, that COPD admissions have stronger associations with PM_2.5_ concentrations after a delay of a few days.

## 4. Discussion

The present study investigated the effects of atmospheric fine particles (PM_2.5_) on hospital admissions for COPD in Southwestern Taiwan. The results showed that PM_2.5_ had a positive association with hospital admissions for COPD in both spring and winter seasons, with different magnitudes in terms of the relative risks and the number of lag days. In addition, the results indicated that PM_2.5_-induced effects varied by season. The observed seasonal differences on PM_2.5_ concentrations and effect estimates might be explained by variations in meteorological parameters, the chemical composition of PM_2.5_ and exposure patterns [23,33,34]. Sea-land breezes (SLBs) that prevail in summer bring abundant moisture, which could reduce the concentrations of atmospheric particles in southwestern Taiwan. The effects of SLBs and the Northeastern Monsoon (NEW) on the spatial and temporal distribution of atmospheric air pollutants over the region of southwestern Taiwan could possibly be explained by the observed seasonal differences in PM_2.5_ levels and chemical constituents in the current study.

In this study, increased risks of COPD admissions associated with ambient PM_2.5_ levels in the spring and winter seasons were observed. This finding is in accordance with a previous Taiwan study that found adverse effects of PM_2.5_ on COPD admissions only on cool days in Kaohsiung [23]. Our finding is also in accordance with a previous study observing an adverse effect on hospital admissions for COPD in Hong Kong, especially during the winter season [10]. However, the prior work by Tsai *et al.* [22] assessed the relative risk of inpatient admissions for COPD in Taipei, Taiwan, and observed an increased risk of COPD admissions associated with ambient PM_2.5_ levels on both warm and cool days. The difference in the effect of PM_2.5_ on COPD admissions in different seasons might be due to the variations in PM_2.5_ chemical constituents, PM_2.5_ levels and behavior or exposure patterns. People in Taipei are more likely to go outdoors and open the windows in the warm season than in the cool season. However, in southwestern Taiwan, summer and autumn seasons are generally hot (the mean temperature was 28 °C) and humid, and most people spend more time indoors. This might explain the reduced effects of PM_2.5_ exposure in patients with COPD in summer in southwestern Taiwan. Nevertheless, the seasonal pattern of PM effects on respiratory diseases requires further study.

Furthermore, this study has provided additional information on the lag effect of PM_2.5_ on COPD admissions, which was not addressed in the previous studies in Taiwan [22,23]. The present study found evidence of a positive linkage between PM_2.5_ levels and COPD hospital admissions for no lag days (Lag 0) up to five days prior to hospitalization, with the most statistically-significant effect being at Lags 0–5. The lag pattern of PM_2.5_ on COPD admissions in the current study was similar to that reported in the Hong Kong study [10]. Kim *et al.* [35] explored the lag structure of association between PM_2.5_ constituents and hospital admissions in Denver, Colorado, and also found that larger effect estimates for respiratory hospital admissions tended to occur at longer lags. The lag effect of PM_2.5_ on respiratory hospitalizations may vary by PM_2.5_ constituents. PM_2.5_ chemical constituents are varied by pollution source and season. Particulate matter with elemental and organic carbon showed more obvious immediate effects than did sulfate and nitrate [35]. Further studies are needed and will be helpful to clarify the temporal lag effect.

Li *et al.* (2015) indicated that short-term exposure to the 10 μg/m^3^ increment of daily ambient PM_2.5_ is associated with a 3.1% (1.6%–4.6%) and a 2.5% (1.5%–3.5%) increase in COPD hospitalizations and COPD mortality, respectively [20]. A study in Beijing, China, by Li Pei *et al.* reported that an increase of 10 μg/m^3^ PM_2.5_ results in an elevation of 0.69% and 1.32% with a lag of three days for respiratory mortality and morbidity, respectively [36]. Lu *et al.* (2015) conducted a meta-analysis in the Chinese population and reported that a 10 μg/m^3^ increase in PM_2.5_ was associated with a 0.75% (95% CI: 01.39%–1.11%) increase in mortality due to respiratory disease [19]. Ko *et al.* conducted a study in Hong Kong and reported that the relative risk for COPD admissions for every 10 μg/m^3^ increase in PM_2.5_ was 1.031 (with a cumulative lag of 0–5) [10]. Compared to other studies in Asia, this study observed larger effect estimates per unit increase of PM_2.5_. The relative risks for COPD admissions for every 10 μg/m^3^ increase in PM_2.5_ at lag zero days were 1.25 (in spring) and 1.24 (in winter), respectively. One potential explanation for this discrepancy might be that most published studies lack effect estimates stratified by season. Kim *et al.* (2012) found that chemical constituents of particulate matter air pollution have seasonal variations in Denver, USA [35]. He *et al.* (2006) investigated the organic components in PM_2.5_ in the urban atmosphere in Beijing and found that different organic compounds presented apparently different seasonal characteristics [37]. Seasonal variations on particulate matter (PM) with different physicochemical constituents might be reflected by increased variability in PM toxicity.

The mean and median levels of PM_2.5_ in this current study were 39.37 and 37.16 μg/m^3^, respectively, compared to the levels of 29.99 and 27.06 μg/m^3^ in Taipei (the largest metropolitan city in Taiwan, located in northern Taiwan) in 2006–2010 [22]. The mean and median levels of PM_2.5_ in this current study were also higher than those in Hong Kong (mean and median levels of 35.7 and 31.7 μg/m^3^) in 2000–2004 [33]. Furthermore, the mean of daily hospital admissions for COPD in the current study was high (8.73) when compared to other administrative districts in Taiwan; for example, the mean of daily hospital admissions for COPD in Kaohsiung (the second largest metropolitan city in Taiwan and situated on the southwest coast of Taiwan) was 7.74 [22]. Some studies have reported the effects of particulate air pollution exposure in patients with respiratory diseases in Taiwan, including Taipei city and Kaohsiung city [22,23]. However, the effects of PM_2.5_ on COPD and their seasonal variations in southwestern Taiwan have never been reported up to the present. In summary, findings from the present study can be helpful to illustrate the health effects of PM_2.5_ on respiratory diseases in Taiwan and to alert the government to pay closer attention to managing air pollution.

Exposure measurement error is an inherent limitation of epidemiology studies of disease and the environment [38]. In the current study, we used PM_2.5_ data from ambient air monitoring sites to represent individual exposure to fine particulate matter. Previous studies have suggested that exposure misclassification from using stationary PM data might lead to diminishing the accuracy of exposure-response estimates compared to personal exposures, thus potentially weakening associations with stationary PM [22,23,38].

Several potential limitations should be taken into consideration. Fist, this study analyzed medical claims data during 2008–2010, and it is too relatively short term to observe the temporal trend for PM_2.5_ effects on COPD. Second, other personal risk factors for COPD hospital admissions, including smoking habit, socioeconomic status and the detailed information, such as the data of pulmonary function tests, were not available from the NHIRD dataset in the current study. Third, personal PM_2.5_ exposure pattern and levels may affect personal exposure and were not taken into account in this study.

## 5. Conclusions

In conclusion, atmospheric fine particulate matter (PM_2.5_) has an association and lag effect on hospital admissions for COPD in Southwestern Taiwan, especially during both spring and winter seasons. Seasonal variation of the PM_2.5_ exposure patterns and levels may play an important role to induce the variations of effect estimations on COPD. Further long-term and large-scale studies are required to analyze the temporal trends on the effects of PM_2.5_ for respiratory diseases in Taiwan.

## Figures and Tables

**Table 1:** Hospitalization rates of chronic obstructive pulmonary disease (COPD) in southwestern Taiwan, 2008–2010.

Hospitalization Rates, /100					
Variables		Total	Male	Female	Male/Female, Hospitalization Rates Ratio
Year	2008	0.19	0.28	0.10	2.80
	2009	0.17	0.24	0.10	2.40
	2010	0.18	0.26	0.10	2.60
	2008–2010 (average)	0.18	0.26	0.10	2.60
Age, year	40–49	0.01	0.02	0.01	2.00
	50–59	0.04	0.07	0.02	3.50
	60–69	0.15	0.24	0.07	3.43
	≥70	0.74	1.14	0.40	2.85
	All	0.18	0.26	0.10	2.60

**Table 2:** Distribution of daily COPD admissions and atmospheric fine particulate matter (PM_2.5_) in southwestern Taiwan, 2008–2010.

Year	Variable ^a^	Min	Percentile			Max	Mean	SD	Days
			25%	50%	75%				
2008	PM_2.5_ (μg/m^3^)	7.84	24.74	38.74	54.20	147.96	41.29	20.68	366
	COPD admissions	0	6	8	11	29	8.89		366
2009	PM_2.5_ (μg/m^3^)	10.71	25.94	38.90	49.65	100.34	40.01	17.24	365
	COPD admissions	0	6	8	11	22	8.63		365
2010	PM_2.5_ (μg/m^3^)	9.54	21.47	35.06	48.06	104.64	36.81	18.79	365
	COPD admissions	0	5	8	11	32	8.67		365
2008–2010 (average)	PM_2.5_ (μg/m^3^)	7.84	24.25	37.16	50.54	147.96	39.37	19.03	1096
	COPD admissions	0	6	8	11	32	8.73	4.50	1096

Abbreviations: Min, minimum value; Max, maximum value; SD, standard deviation; ^a^ 24-h average.

**Table 3:** Relative risks (with 95% CI) for the atmospheric fine particulate matter (PM_2.5_) per 10 μg/m^3^ increase in the concentration of PM_2.5_ for COPD admissions in southwestern Taiwan, 2008–2010.

COPD Admissions								
Lag Days	Spring (February–April)		Summer (May–July)		Autumn (August–October)		Winter (November–January)	
	PM_2.5_^a^ = 48.54 μg/m^3^		PM_2.5_ = 25.89 μg/m^3^		PM_2.5_ = 33.37 μg/m^3^		PM_2.5_ = 49.96 μg/m^3^	
	RR ^a^	95% CI	RR	95% CI	RR	95% CI	RR	95% CI
Lag 0	1.25 **	(1.22, 1.27)	0.53 **	(0.51, 0.55)	0.81 **	(0.79, 0.82)	1.24 **	(1.23, 1.26)
Lags 0–1	1.30 **	(1.27, 1.33)	0.52 **	(0.50, 0.53)	0.77 **	(0.76, 0.79)	1.30 **	(1.28, 1.32)
Lags 0–2	1.35 **	(1.32, 1.38)	0.50 **	(0.48, 0.52)	0.75 **	(0.73, 0.77)	1.36 **	(1.34, 1.39)
Lags 0–3	1.40 **	(1.37, 1.44)	0.48 **	(0.47, 0.50)	0.73 **	(0.71, 0.75)	1.42 **	(1.39, 1.45)
Lags 0–4	1.46 **	(1.42, 1.50)	0.48 **	(0.46, 0.50)	0.71 **	(0.69, 0.73)	1.48 **	(1.45, 1.51)
Lags 0–5	1.51 **	(1.46, 1.55)	0.47 **	(0.46, 0.49)	0.69 **	(0.67, 0.71)	1.54 **	(1.50, 1.58)

Abbreviation: RR, relative risk; **, *p* < 0.01; ^a^ 24-h average concentrations.
